# Pioglitazone Enhances Mitochondrial Biogenesis and Ribosomal Protein Biosynthesis in Skeletal Muscle in Polycystic Ovary Syndrome

**DOI:** 10.1371/journal.pone.0002466

**Published:** 2008-06-18

**Authors:** Vibe Skov, Dorte Glintborg, Steen Knudsen, Qihua Tan, Thomas Jensen, Torben A. Kruse, Henning Beck-Nielsen, Kurt Højlund

**Affiliations:** 1 Department of Biochemistry, Genetics, and Pharmacology, Odense University Hospital and Human Microarray Centre (HUMAC), University of Southern Denmark, Odense, Denmark; 2 Diabetes Research Centre, Department of Endocrinology, Odense University Hospital, Odense, Denmark; 3 Medical Prognosis Institute Aps, Hørsholm, Denmark; 4 Institute of Public Health, University of Southern Denmark, Odense, Denmark; University of Cape Town, South Africa

## Abstract

Insulin resistance is a common metabolic abnormality in women with PCOS and leads to an elevated risk of type 2 diabetes. Studies have shown that thiazolidinediones (TZDs) improve metabolic disturbances in PCOS patients. We hypothesized that the effect of TZDs in PCOS is, in part, mediated by changes in the transcriptional profile of muscle favoring insulin sensitivity. Using Affymetrix microarrays, we examined the effect of pioglitazone (30 mg/day for 16 weeks) on gene expression in skeletal muscle of 10 obese women with PCOS metabolically characterized by a euglycemic-hyperinsulinemic clamp. Moreover, we explored gene expression changes between these PCOS patients before treatment and 13 healthy women. Treatment with pioglitazone improved insulin-stimulated glucose metabolism and plasma adiponectin, and reduced fasting serum insulin (all *P*<0.05). Global pathway analysis using Gene Map Annotator and Pathway Profiler (GenMAPP 2.1) and Gene Set Enrichment Analysis (GSEA 2.0.1) revealed a significant upregulation of genes representing mitochondrial oxidative phosphorylation (OXPHOS), ribosomal proteins, mRNA processing reactome, translation factors, and proteasome degradation in PCOS after pioglitazone therapy. Quantitative real-time PCR suggested that upregulation of OXPHOS genes was mediated by an increase in PGC-1α expression (*P*<0.05). Pretreatment expression of genes representing OXPHOS and ribosomal proteins was down-regulated in PCOS patients compared to healthy women. These data indicate that pioglitazone therapy restores insulin sensitivity, in part, by a coordinated upregulation of genes involved in mitochondrial OXPHOS and ribosomal protein biosynthesis in muscle in PCOS. These transcriptional effects of pioglitazone may contribute to prevent the onset of type 2 diabetes in these women.

## Introduction

Polycystic ovary syndrome (PCOS) is a common endocrine and metabolic disorder occurring in 5–10% of premenopausal women. Symptoms of PCOS include menstrual irregularities, hyperandrogenism, and infertility [1]. Several studies have shown that insulin resistance plays an important role in the pathogenesis of PCOS [2] and increases the risk for development of type 2 diabetes [1], [2]. Thiazolidinediones (TZDs), including pioglitazone, are peroxisome proliferator-activated receptor (PPAR)-γ agonists that induce adipogenesis and have insulin-sensitizing and antidiabetic properties [3]. TZDs operate via receptor-dependent or independent mechanisms thereby regulating the expression of genes involved in mitochondrial biogenesis, insulin signal transduction, and glucose and lipid metabolism [4], [5]. PPARγ is most abundantly expressed in adipose tissue and to a lesser extent in muscle and liver tissue [6].

Recent studies have demonstrated that the beneficial metabolic effects of treatment with pioglitazone in PCOS patients partly occur through improvement of insulin sensitivity, including increased insulin-stimulated total, oxidative and non-oxidative glucose transport, and a decrease in insulin secretion [7], [8], similar to observations in patients with type 2 diabetes [9]. Skeletal muscle accounts for the majority of insulin-stimulated glucose transport suggesting an important role for TZDs in this tissue.

The mechanisms by which TZDs exert their insulin sensitizing action in skeletal muscle of type 2 diabetic patients are not yet fully understood, but may include increased downstream insulin receptor signaling [10], [11] and enhanced fatty acid uptake and oxidation [12], [13]. Moreover, animal studies have demonstrated that prolonged treatment with TZDs is associated with increased AMP-activated protein kinase (AMPK) activity [14], and increased expression of NADH dehydrogenase subunit 1 of complex I and PPAR-γ coactivator-1α (PGC-1α) in skeletal muscle [15]. These observations suggest that TZDs may increase insulin sensitivity, in part, by improving mitochondrial oxidative metabolism (OXPHOS). We have recently demonstrated reduced expression of genes involved in OXPHOS in skeletal muscle of insulin resistant women with PCOS [16]. However, to our knowledge, no study has examined whether changes in muscle transcripts contribute to the insulin sensitizing effect of TZDs in PCOS patients.

DNA microarrays are high throughput technologies aiming at measuring transcript abundance for thousands of genes simultaneously [17]. To explore mRNA levels in diseases where differences in gene expression are too modest or numerous to extract meaningful biological function from individual genes, the application of global pathway analysis has been promising in detecting coordinated changes in gene expression levels [16], [18]–[20].

In the present study, we hypothesized that pioglitazone treatment may modify the expression of pathways representing OXPHOS genes as well as other pathways implicated in skeletal muscle insulin resistance of PCOS patients. We used two different approaches for global pathway analysis, and quantitative real-time PCR (q-RT-PCR) was applied to evaluate microarray results.

## Results

### Clinical and metabolic characteristics

After treatment with pioglitazone, basal serum insulin levels were reduced, whereas plasma adiponectin increased 2-fold (all *P*<0.01) (Table 1). No significant changes in plasma triglycerides, basal plasma glucose, serum free testosterone, or plasma FFA levels were observed. Insulin-stimulated total glucose disposal was increased by 36% in PCOS patients in response to pioglitazone (*P*<0.01), and this was partly accounted for by a 26% increase in glucose oxidation (*P*<0.05) and a 50% increase in non-oxidative glucose metabolism (*P*<0.01). There was no significant change in the ability of insulin to suppress lipid oxidation in response to pioglitazone.

**Table 1:** Clinical and metabolic characteristics of PCOS patients and control subjects.

	Control Subjects	PCOS Pretreatment	PCOS Posttreatment
*n*	13	10	10
Age (years)	34.7±2.0	30.3±2.1	
Body mass index (kg/m^2^)	34.0±1.8	33.2±0.9	33.0±1.1
Body fat (%)	41±1.6	39.1±1.3	39.8±1.4
Plasma triglycerides (mmol/l)	0.86±0.12 †	1.43±0.22	1.15±0.16
Serum free testosterone (nmol/l)	0.025±0.003 ††	0.053±0.009	0.048±0.007
Plasma glucose basal (mmol/l)	5.6±0.1	5.9±0.2	5.6±0.1
Serum insulin basal (pmol/l)	51±6 ††	125±23	69±12††
Plasma FFA basal (mmol/l)	0.49±0.04	0.45±0.05	0.41±0.05
Plasma adiponectin (mg/l)	9.4±0.8	7.1±0.9	16.1±2.2††
Rd basal (mg·min^−1^·m^−2^)	72±3	75±3	76±4
Rd clamp (mg·min^−1^·m^−2^)	290±23 ††	138±18	188±25††
Glucose oxidation basal (mg·min^−1^·m^−2^)	52±8	46±5	43±10
Glucose oxidation clamp (mg·min^−1^·m^−2^)	124±5 ††	80±10	101±12†
Lipid oxidation basal (mg·min^−1^·m^−2^)	33±3	38±1	40±4
Lipid oxidation clamp (mg·min^−1^·m^−2^)	7±2 ††	24±4	16±5
NOGD basal (mg·min^−1^·m^−2^)	20±7	30±5	34±9
NOGD clamp (mg·min^−1^·m^−2^)	165±22 ††	58±12	87±14††

PCOS pretreatment vs. controls, and the effect of pioglitazone treatment (30 mg/day for16 weeks) in PCOS patients. Students *T*-test for non-paired and paired data used, respectively. Data represent means±SEM. ^††^P<0.01 and ^†^P<0.05 vs. PCOS pretreatment. NOGD, non-oxidative glucose disposal.

Pretreatment levels of plasma triglycerides (*P*<0.05), basal serum insulin and free testosterone levels were elevated (*P*<0.01), and plasma adiponectin tended to be decreased (*P*<0.07) in PCOS patients compared with control subjects. There was no significant difference with respect to basal plasma glucose and FFA. Insulin-mediated total, non-oxidative and oxidative glucose metabolism and suppression of lipid oxidation were impaired in PCOS patients compared with control subjects (all *P*<0.01).

### Oligonucleotide microarray analysis

Statistical analysis of the 54.675 probe sets represented on the array revealed that 23.933 probe sets were differentially regulated in PCOS patients in response to treatment with pioglitazone (uncorrected *P*<0.05). 5.303 probe sets remained significantly differentially expressed when corrected for multiple testing using the Benjamini-Hochberg method (FDR<0.01) [21].

Pretreatment levels of 14.834 probe sets were differentially expressed in PCOS patients compared with control subjects. After correction for multiple testing (FDR<0.05), 2.754 probe sets remained significantly differentially expressed [21]. To examine this huge amount of regulated transcripts, we decided to apply global pathway analysis to identify significantly regulated pathways.

### Effects of pioglitazone evaluated by global pathway analysis

The pathways that were regulated in skeletal muscle of PCOS patients in response to pioglitazone treatment were examined by Gene Map Annotator and Pathway Profiler (GenMAPP 2.1) together with its accessory program, MAPPFinder 2.1, and by gene set enrichment analysis (GSEA 2.0.1). Using MAPPFinder, the top ranked upregulated pathways were *ribosomal proteins*, *electron transport chain*, *mRNA processing reactome*, *ubiquinone biosynthesis*, *proteasome degradation*, and *translation factors* (family wise error rate (FWER) <0.05) (Table 2). Applying GSEA, the *electron transport chain*, *VOXPHOS*, *insulin 2F up* (genes 2 fold upregulated by insulin), *ribosomal proteins*, *mRNA splicing*, *mRNA processing*, *circadian exercise* (genes that regulate the 24-hour cycle), *rapamycin_DN*, *glutamine_DN* (genes downregulated in response to rapamycin or glutamine starvation, respectively), *mRNA processing reactome*, *oxidative phosphorylation*, *translation factors*, *Human MitoDB 6*, *proteasome degradation*, and *leucine_DN* (genes downregulated in response to leucine starvation) were significantly upregulated (FWER<0.05) (Table 3). A further description of gene sets from GSEA can be obtained from the GSEA homepage [22]. No pathways or gene sets were significantly downregulated (FWER<0.05) when using MAPPFinder or GSEA (Table S1 and S2).

**Table 2:** The top-ten most upregulated pathways analyzed with MAPPFinder 2.1.

MAPP Name	Changed (n)	Measured (n)	ON MAPP (n)	Changed (%)	Z Score	Permute p-value	FWER p-value
Ribosomal proteins	69	88	88	78.4	10.9	<0.0005	<0.0005
Electron transport chain	61	91	105	67.0	8.6	<0.0005	<0.0005
mRNA processing reactome	62	125	127	49.6	5.7	<0.0005	<0.0005
Ubiquinone biosynthesis	32	54	81	59.3	5.3	<0.0005	<0.0005
Proteasome degradation	31	60	61	51.7	4.3	<0.0005	0.003
Translation factors	26	50	50	52.0	4.0	<0.0005	0.02
TGF beta receptor netpath 7	57	151	151	37.7	3.0	0.003	0.43
Striated muscle contraction	18	38	38	47.4	2.8	0.007	0.56
Androgen receptor netpath 2	43	112	112	38.4	2.7	0.008	0.65
TNF alpha NFkB netpath 9	66	186	187	35.5	2.6	0.005	0.75

A p-value<0.05 and a fold change ≥1.05 were used as the criteria for gene expression changes in PCOS patients after pioglitazone treatment. The z-score is based on N = 4998 genes linked to a MAPP and R = 1355 of these genes meeting the criteria for change in expression. Changed (n): number of genes changed. Measured (n): number of genes measured on the chip. On MAPP (n): number of genes on the MAPP. Changed (%): Changed (n) divided by Measured (n). FWER p-value: Family Wise Error Rate.

**Table 3:** Ranking of the 15 most upregulated gene sets analyzed with GSEA 2.0.1.

Name	Size	ES	NES	NOM p-value	FDR q-value	FWER p-value
Electron transport chain	97	−0.60	−3.00	<0.0001	<0.0001	<0.0001
VOXPHOS	77	−0.62	−3.00	<0.0001	<0.0001	<0.0001
Insulin 2F up	188	−0.52	−2.89	<0.0001	<0.0001	<0.0001
Ribosomal proteins	88	−0.53	−2.61	<0.0001	<0.0001	<0.0001
mRNA splicing	48	−0.56	−2.43	<0.0001	<0.0001	<0.0001
mRNA processing	42	−0.57	−2.36	<0.0001	<0.0001	<0.0001
Circadian exercise	42	−0.56	−2.32	<0.0001	0.0002	0.0005
Rapamycin DN	189	−0.40	−2.19	<0.0001	0.0007	0.002
Glutamine DN	251	−0.38	−2.12	<0.0001	0.001	0.004
mRNA processing reactome	108	−0.40	−2.01	<0.0001	0.003	0.01
Oxidative phosphorylation	58	−0.44	−1.97	<0.0001	0.004	0.01
Translation factors	47	−0.46	−1.96	<0.0001	0.004	0.02
Human MitoDB 6 2002	385	−0.32	−1.95	<0.0001	0.005	0.02
Proteasome degradation	31	−0.50	−1.93	<0.0001	0.005	0.02
Leucin DN	141	−0.36	−1.92	<0.0001	0.005	0.03

Gene sets with a FWER<0.05 are shown. All genes on the chip were ranked by difference in expression after pioglitazone treatment of PCOS patients using the t-test. An enrichment score (ES) was assigned to each gene, and the maximum ES (MES) was calculated for each gene set. NES: Enrichment score normalized for differences in gene set size. FDR q-value: False Discovery Rate. FWER p-value: Family Wise Error Rate.

Using GO, *ribonucleoprotein complex* and *ribosome* were the most upregulated cellular components (C), and *structural constituent of ribosome* was the most upregulated molecular function (F) term. The most upregulated biological process (P) term was *protein biosynthesis*. *Oxidoreductase activity, acting on NADH or NADPH and NADH dehydrogenase activity* was among the top twenty upregulated GO terms (Table S3). *Signal transducer activity* was the most downregulated molecular function (F) term, and *cell communication* was the most downregulated biological process (P) term. Membrane and related terms were the most downregulated cellular components (C) (Table S4).

Evaluating the results from both GenMAPP and GSEA, pathways representing OXPHOS genes, *ribosomal proteins*, *mRNA processing reactome*, *translation factors*, and *proteasome degradation* were significantly upregulated (FWER<0.05).

### Pretreatment abnormalities in PCOS examined by global pathway analysis

To test whether the transcriptional changes induced by pioglitazone were correcting preexisting abnormalities in patients with PCOS, MAPPFinder 2.1 and GSEA 2.0.1 were also applied for analysis of gene expression changes between 10 PCOS patients before treatment and 13 control subjects. Pretreatment muscle biopsies from seven of the 10 PCOS patients analyzed in the present study were also included in a previous microarray study, in which the 16 most insulin resistant of all larger cohort of PCOS patients [7] were compared with the same 13 control subjects [16]. In MAPPFinder, *ribosomal proteins* and the *electron transport chain* were significantly downregulated (FWER<0.05) (Table 4). Using GSEA, the expression of *VOXPHOS*, *electron transport chain*, *valine, leucine, and isoleucine degradation*, *propanoate metabolism*, *oxidative phosphorylation*, *Human mitoDB 6*, *insulin 2F up*, *ribosomal proteins*, and *fatty acid metabolism* was significantly decreased (FWER<0.05) (Table 5). No pathways and gene sets remained significantly upregulated in MAPPFinder and GSEA after correction for multiple testing (FWER) (Table S5 and S6). Applying GO, *cell communication* was the most upregulated biological process (P), *enzyme regulator activity* the most upregulated molecular function (F), and *intrinsic to membrane* the most upregulated cellular component (Table S7). The most downregulated GO terms were *ribosome* and *ribonucleoprotein complex* (C), *structural constituent of ribosome* (F), and *protein biosynthesis* (P) (Table S8).

**Table 4:** The ten most downregulated pathways analyzed with MAPPFinder 2.1.

MAPP Name	Changed (n)	Measured (n)	ON MAPP (n)	Changed (%)	Z Score	Permute p-value	FWER p-value
Ribosomal proteins	43	88	88	48.9	9.2	<0.0005	<0.0005
Electron transport chain	39	91	105	42.9	7.7	<0.0005	<0.0005
Aminoacyl tRNA biosynthesis	10	23	24	43.5	3.9	<0.0005	0.07
Ubiquinone biosynthesis	17	54	81	31.5	3.5	0.002	0.18
Phenylalanine, tyrosine, and tryptophan biosynthesis	5	11	36	45.5	2.9	0.01	0.55
Inositol metabolism	1	1	9	100.0	2.4	0.13	0.96
Translation factors	13	50	50	26.0	2.3	0.02	0.97
mRNA processing reactome	27	125	127	21.6	2.2	0.03	0.98
Cell cycle G1 to S control reactome	16	67	67	23.9	2.2	0.04	0.99
Mitochondrial fatty acid betaoxidation	5	16	16	31.3	1.9	0.08	1
Insulin signaling	31	159	159	19.5	1.8	0.07	1

A p-value<0.05 and a fold change ≤−1.05 were used as the criteria for gene expression changes between PCOS patients and control subjects. The z-score is based on N = 4998 genes linked to a MAPP and R = 730 of these genes meeting the criteria for change in expression. Changed (n): number of genes changed. Measured (n): number of genes measured on the chip. On MAPP (n): number of genes on the MAPP. Changed (%): Changed (n) divided by Measured (n). FWER p-value: Family Wise Error Rate.

**Table 5:** Ranking of the ten most downregulated gene sets analyzed with GSEA 2.0.1.

Name	Size	ES	NES	NOM p-value	FDR q-value	FWER p-value
VOXPHOS	77	0.61	3.06	<0.0001	<0.0001	<0.0001
Electron transport chain	97	0.58	2.99	<0.0001	<0.0001	<0.0001
Valine, leucine, and isoleucine degradation	36	0.55	2.30	<0.0001	<0.0001	<0.0001
Propanoate metabolism	31	0.57	2.29	<0.0001	<0.0001	<0.0001
Oxidative phosphorylation	58	0.46	2.13	<0.0001	0.0007	0.002
Human mitoDB 6 2002	385	0.34	2.13	<0.0001	0.0005	0.002
Insulin 2F up	188	0.35	2.05	<0.0001	0.002	0.005
Ribosomal proteins	88	0.42	2.04	<0.0001	0.002	0.007
Fatty acid metabolism	79	0.40	1.94	<0.0001	0.006	0.03
Cell cycle arrest	32	0.45	1.83	0.005	0.01	0.06

All genes on the chip were ranked by difference in expression between PCOS patients and control subjects using the t-test. An enrichment score (ES) was assigned to each gene, and the maximum ES (MES) was calculated for each gene set. NES: Enrichment score normalized for differences in gene set size. FDR q-value: False Discovery Rate. FWER p-value: Family Wise Error Rate.

Significant downregulation (FWER<0.05) of genes in pathways representing OXPHOS and ribosomal proteins in PCOS patients was identified using both GenMAPP and GSEA, and, in part, validated our previous findings, where we used the same cohort of control subjects and 16 insulin resistant PCOS patients [16].

### Validation of pathway analysis with q-RT-PCR

**Figure 1:**
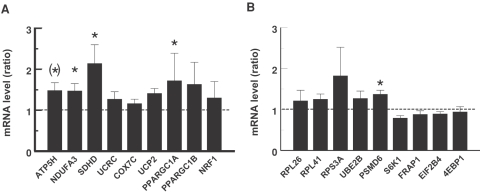
Effect of pioglitazone on expression of muscle genes. The mRNA expression level of selected genes was determined by quantitative real-time PCR in nine patients with the polycystic ovary syndrome (PCOS). The relative expression of genes involved in oxidative phosphorylation (*A*) and ribosomal protein biosynthesis including signaling to protein synthesis (*B*) are given. Regulated genes have mRNA levels different from 1.0 (dotted line). Data are means±SEM. *P<0.05.

The effect of pioglitazone treatment on expression of genes involved in OXPHOS and ribosomal protein synthesis and degradation was validated by q-RT-PCR (Table S9). All five respiratory genes examined and UCP2 showed an increase in expression (1.2–2.1 fold). Of these, NDUFA3 and SDHD were significantly upregulated, and ATP5H tended (*P* = 0.057) to be upregulated (Figure 1*A*). The expression of genes known to mediate transcriptional control of mitochondrial biogenesis, PPARGC1A (PGC-1α), PPARGC1B (PGC-1β) and NRF1, increased 1.4–1.7 fold in response to pioglitazone. However, only the increase in PGC-1α achieved statistical significance (*P*<0.02). The mRNA level of PSMD6 was significantly increased, whereas the increase (1.2–1.8 fold) in expression of the other genes representing protein metabolism, RPS3A, RPL26, RPL41, and UBE2B, was not significant (Figure 1*B*). Moreover, the gene expression of enzymes involved in signaling to protein synthesis including EIF2B4, FRAP1, S6K1 and 4EBP1 was unaltered in response to pioglitazone. We did not evaluate gene expression changes in skeletal muscle between patients with PCOS and control subjects with q-RT-PCR, because these changes have been validated recently [16].

## Discussion

In this study, we examined the molecular signature of pioglitazone therapy in skeletal muscle of women with PCOS using global transcriptional profiling. We demonstrate that increased insulin sensitivity induced by pioglitazone treatment is associated with enhanced expression of genes representing ribosomal protein biosynthesis and OXPHOS pathways, the latter possibly mediated by an increased expression of PGC-1α. Furthermore, we provide evidence that treatment with pioglitazone corrects preexisting downregulation of the same pathways in skeletal muscle of patients with PCOS. These findings suggest that the insulin-sensitizing effect of pioglitazone therapy may include reversal of abnormalities in ribosomal protein biosynthesis and mitochondrial oxidative phosphorylation.

In a recent microarray study [16], we found reduced expression of OXPHOS genes in skeletal muscle biopsies obtained from the most insulin resistant women among a larger cohort of PCOS patients. Here, we confirm the downregulation of OXPHOS genes in skeletal muscle of PCOS patients, who were randomly assigned to treatment with pioglitazone in the same study cohort. In addition, we found reduced expression of the ribosomal protein pathway using both GenMAPP and GSEA. Although this abnormality was not detected in our previous study of PCOS patients [16], reduced gene expression of ribosomal proteins has been reported in skeletal muscle [19] and adipose tissue [23] of patients with type 2 diabetes in microarray-based studies. Moreover, GO analysis showed that protein biosynthesis was the most downregulated biological process term. These findings support the hypothesis of an association between reduced protein synthesis and insulin resistance in skeletal muscle, such as shown in age-related sarcopenia [24], [25]. However, Halvatsiotis *et al* [26] reported no changes in muscle protein synthesis rate in patients with type 2 diabetes, indicating that transcriptional downregulation of the translational machinery is not necessarily reflected by a decreased protein synthesis rate. Thus, whether muscle protein synthesis rate is affected in PCOS patients remains to be determined, but our data indicate that reduced gene expression of ribosomal proteins and other pathways involved in protein metabolism can not be attributed to hyperglycemia or obesity, and, therefore, may represent an early defect associated with insulin resistance in PCOS.

In the present study, the major finding is that the expression of genes representing OXPHOS pathways is upregulated in skeletal muscle of PCOS patients together with an increase in insulin sensitivity in response to 16 weeks treatment with pioglitazone. To our knowledge, no study has addressed the effects of TZDs on muscle transcripts in PCOS. Similar to other studies using pathway analysis [16], [18], [19], we observed small, but significant, changes in expression of many genes representing OXPHOS pathways, and some of these changes were also confirmed by q-RT-PCR. Thus, these data provide evidence for an association between small, but coordinated, improvements in expression of OXPHOS genes in skeletal muscle and insulin sensitivity after TZD treatment in women with PCOS. In a recent report, Pagel-Langenickel *et al* [27] showed that improved glycemic control in patients with type 2 diabetes treated with rosiglitazone for 12 weeks was independent of muscle mitochondrial content/activity. Thus, rosiglitazone-mediated improvement in OXPHOS protein content and citrate synthase activity was observed only in individuals with relatively preserved maximal oxygen consumption and mitochondrial copy numbers. Indeed, the difference between our study and the study by Pagel-Langenickel *et al* [27] may reflect a modestly longer duration of therapy (16 vs. 12 weeks), and the broader PPAR isoform activation activity of pioglitazone versus other TZDs such as rosiglitazone. Another possibility is that intervention with TZDs earlier in the spectrum of insulin resistance, e.g. in normoglycemic patients with PCOS, has a greater effect on improving mitochondrial biogenesis than in patients with type 2 diabetes. Moreover, expression of muscle mRNA may not accurately reflect the abundance or activity of OXPHOS proteins, and further studies are warranted to establish whether improved OXPHOS gene expression in response to TZDs is associated with increased content and function of muscle mitochondria in PCOS.

PPARγ ligands such as TZDs may enhance skeletal muscle insulin sensitivity by inducing expression of genes like adiponectin or genes involved in fatty acid uptake and oxidation in adipose tissue which, in turn, convey signals to skeletal muscle [4], [28]. Thus, recombinant adiponectin stimulates fatty acid oxidation and glucose transport by activation of AMPK in rodent muscle [29], [30]. Other studies have shown that TZDs also may exert their action independently of PPARγ [31] and adiponectin, e.g. by direct activation of AMPK [32], [33]. Chronic activation of AMPK is associated with increased mitochondrial biogenesis [34), probably mediated by increased expression of PGC-1α and NRF-1 [34], [35]. In a single study of skeletal muscle from type 2 diabetic patients, Bandyopadhyay *et al* [12] showed that chronic rosiglitazone therapy restored total AMPK activity. Contradictory to this, the activity and protein content of AMPK in muscle were not increased in our pioglitazone treated PCOS subjects, despite a 2-fold increase in circulating adiponectin [36]. This may indicate that other mechanisms independently of AMPK mediated the effect of TZDs on PGC-1α transcription, mitochondrial biogenesis and insulin sensitivity in PCOS, e.g. through calcium/calmodulin-dependent protein kinase and p38 mitogen-activated protein kinase [37], [38]. Recently, it was shown that the effects of AMPK on gene expression of glucose transporter 4, mitochondrial genes and PGC-1α, are mediated almost entirely by phosphorylation of PGC-1α at specific sites [39]. Thus, another possibility is that pioglitazone treatment of women with PCOS causes a transient increase in AMPK activity, which then disappears in response to enhanced OXPHOS gene expression and ATP synthesis.

Downregulation of PGC-1α is associated with reduced expression of OXPHOS genes in skeletal muscle of women with PCOS [16], patients with type 2 diabetes [18], [19], and their first-degree relatives [19]. In the current study, treatment with pioglitazone increased the expression of PGC-1α in muscle of PCOS patients providing an explanation at the molecular level for the observed upregulation of OXPHOS genes. These data are consistent with a recent study showing improved skeletal muscle oxidative enzyme activity and restoration of PGC-1α gene expression upon rosiglitazone treatment in obese patients with type 2 diabetes [40]. Whether the effect on PGC-1α is mediated by the 2-fold increase in adiponectin, a direct effect of pioglitazone on PPARγ, or other as yet unknown effects of pioglitazone remains to be clarified.

Another mechanism by which pioglitazone may enhance OXPHOS gene expression in PCOS is via the effect of insulin on mitochondrial biogenesis. Thus, studies have shown that acute and chronic insulin infusion enhances the transcriptional activity of genes involved in pathways such as energy metabolism in skeletal muscle of healthy subjects and patients with type 2 diabetes [41]–[43]. In the present study, a pathway termed Insulin 2F Up, representing genes upregulated 2-fold by insulin stimulation of skeletal muscle in healthy subjects [41], was significantly upregulated in response to pioglitazone. Thus, it is possible that the effect of pioglitazone on OXPHOS gene expression is, at least in part, mediated by an improved insulin action in muscle of PCOS patients. However, further studies are needed to address the exact molecular mechanisms by which pioglitazone treatment improves insulin action on transcriptional activity in women with PCOS.

Using two different approaches for global pathway analysis, we observed a consistent coordinated up-regulation of genes involved in ribosomal protein biosynthesis in muscle of PCOS patients after pioglitazone administration. A similar effect of TZDs on muscle protein metabolism has, to our knowledge, not been reported previously in PCOS or other insulin resistant conditions. Our data indicate that the beneficial effects of pioglitazone therapy in PCOS may include improved protein metabolism mediated either via an enhanced anabolic action of insulin or directly via PPARγ by an as yet unknown mechanism. Several lines of evidence support a role for increased insulin action. Thus, insulin promotes protein synthesis by rapidly activating several components of the translational machinery [44]. This response is elicited primarily through phosphoinositide 3-kinase (PI3K) and Akt, which via inhibition of GSK-3 causes dephosphorylation and activation of eIF2B, and via activation of the mammalian target of rapamycin (mTOR) signaling and phosphorylation of its downstream targets ribosomal S6 kinase 1 (S6K1) and activation of eukaryotic initiation factor 4E binding protein 1 (4EBP1), promotes initiation and elongation [44]. Although our data provide evidence that the increased transcription of ribosomal proteins was not caused by changes in the expression of these key components in insulin signaling to protein synthesis, we have recently reported that impaired insulin action on Akt phosphorylation in muscle of PCOS patients was normalized after treatment with pioglitazone [36]. Thus, changes in the activity of mTOR signaling enzymes and eIF2B provides a possible explanation for the observed changes in the translational machinery before and after pioglitazone treatment. Moreover, recent microarray-based studies of gene expression in skeletal muscle of healthy humans have shown that the majority of the genes upregulated in response to acute insulin infusion codes for proteins involved in transcriptional and translational regulation including a number of ribosomal proteins [41], [45]. These reports lend support to the hypothesis that in the long term, insulin also increases the cellular content of ribosomes augmenting the capacity for protein synthesis. Interestingly, there is experimental evidence that mTOR activity may also play a regulatory role for mitochondrial metabolism [46]. Thus, improved expression of genes involved in ribosomal protein biosynthesis and mitochondrial biogenesis in skeletal muscle of PCOS patients after long term pioglitazone therapy may involve enhanced mTOR signaling mediated in part by an improved anabolic action of insulin.

In summary, pioglitazone significantly upregulated a large number of pathways in skeletal muscle of women with PCOS. The most important finding was that pioglitazone increased the expression of genes representing OXPHOS and ribosomal protein biosynthesis, and that this effect corrected preexisting downregulation of genes in the same pathways. Upregulation of OXPHOS genes seems to involve increased expression of PGC-1α, and may occur via an increase in adiponectin, a direct effect of TZD on muscle PPAR gamma, and/or via an improved insulin action on mitochondrial biogenesis. Further studies are required to assess the precise mechanisms by which prolonged treatment with TZD increases expression of OXPHOS and ribosomal protein biosynthesis in skeletal muscle, and how these transcriptional changes improves insulin sensitivity.

## Materials and Methods

### Subjects

Ten obese Caucasian women of reproductive age with PCOS participated in the study to test the effect of pioglitazone therapy on skeletal muscle gene expression. These subjects represent all women with PCOS from whom a muscle biopsy was obtained both before and after treatment with pioglitazone among a larger cohort of PCOS patients [7], [16]. Briefly, thirty PCOS patients were randomly assigned to either pioglitazone or placebo. No effect with respect to hormonal or metabolic parameters was found in the placebo group after treatment cessation.

Criteria for PCOS included irregular periods with cycle length >35 days during the last year, free testosterone level above reference interval (>0.035 nmol/l), and/or hirsutism (total Ferriman-Gallwey score>7) [7]. All PCOS patients accepted to withdraw oral contraceptives >3 months before evaluation and consented to use barrier contraception combined with spermatocidal cream during the study period. Control subjects had regular menses, normal glucose tolerance, and no family history of diabetes. Women with diabetes (fasting plasma glucose≥7.0 mmol/l), hypertension, elevated liver enzyme levels, adrenal enzyme defects, hyperprolactinemia, and hypothyroidism were excluded from the study. Participants were excluded if they were pregnant. All participants had normal results on screening blood tests of hepatic and renal function. No subjects were taking any medication known to affect hormonal or metabolic parameters. Informed written consent was obtained from all subjects before participation. The study was approved by the Local Ethics Committee and was performed in accordance with the Helsinki Declaration.

### Study design

The design of the study has previously been described [7]. All subjects were instructed to refrain from strenuous physical activity for a period of 48-h before the euglycemic-hyperinsulinemic clamp studies. The study subjects were admitted to the Diabetes Research Centre at Odense University Hospital at 08:00 after an overnight fast. To test the effect of a PPAR-γ agonist, pioglitazone (30 mg/day; Actos, Takeda, Lilly A/S, Lyngby, Denmark) or placebo was administered to PCOS patients for a period of 16 weeks. One patient receiving pioglitazone was excluded from the study because of side effects (dizziness, ankle edema, and anxiety). After the treatment period, the initial evaluation program was repeated [7].

Rates of total glucose disposal rates (Rd), glucose and lipid oxidation, and non-oxidative glucose disposal (NOGD) were assessed by euglycemic-hyperinsulinemic clamp studies (4-h insulin infusion, 40 mU7min per m^2^) combined with indirect calorimetry as described in detail previously [7]. Skeletal muscle biopsies were obtained in the basal steady-state period of the clamp from the vastus lateralis muscle using a modified Bergström needle with suction under local anaesthesia (10–15 ml of lidocain 2% (20 g/l)). Muscle biopsies were immediately blotted free of blood, fat, and connective tissue and frozen in liquid nitrogen within 30 s. Assays for serum levels of insulin, free testosterone, luteinizing hormone (LH), follicle-stimulating hormone (FSH), plasma glucose, triglyceride, and free fatty acids (FFA) were as described in [7].

### RNA extraction and microarray preparation

Total RNA was extracted from skeletal muscle tissue using the TRIzol protocol (Life Technologies, Gaithersburg, MD). An extra phenol-chloroform step was included as described previously [16]. Quantity of RNA was determined with a spectrophotometer, and RNA of high quality was assessed using Agilent 2100 Bioanalyser (Agilent Technologies, Palo Alto, CA) and degradometer software [47]. The MessageAmpTM II-Biotin single round aRNA amplification kit (Ambion, Austin, TX) was applied to convert one µg of purified total RNA to biotin-labeled aRNA. Labeled aRNA was fragmented as described in the Affymetrix manual (Affymetrix, Santa Clara, CA) and hybridized to Affymetrix HG-U133 Plus 2.0 chips. The 3′/5′ ratio of Glyceraldehyde-3-phosphate dehydrogenase (GAPDH) was below 1.42 for all chips and confirmed high quality RNA.

### Data processing

The R statistical software [48] was applied for data preprocessing (www.bioconductor.org) and statistical analysis. Global background correction of probe intensities was done by employing a method implemented in the robust multi-array average (RMA) method [49], and data was normalized by using constant normalization. We tried to use variance stabilizing transformation (VSN) for normalization as well. The results were comparable to those obtained using constant normalization (data not shown). Gene expression index calculation was done using model based index calculation (MBEI) by Li & Wong [50]. Only perfect match probes were included in data analysis. Differences in gene expression between PCOS patients before and after pioglitazone therapy and between PCOS patients and controls were calculated for each gene by using Welch two sample t-test for paired and unpaired data, respectively. An uncorrected *P*<0.05 was considered significant. Data are available from GEO (http://www.ncbi.nlm.nih.gov/geo, Accession No. GSE8157).

### Global pathway analysis

GenMAPP 2.1 [51] (MAPPFinder 2.1 (20)), and GSEA 2.0.1 [52] were employed to assess significantly regulated pathways, gene ontology (GO) terms, and gene sets in the two data sets. In MAPPFinder, a total of 203 pathways and 6251 GO terms were applied whereas 189 gene sets were used in GSEA. An enrichment score (ES) was calculated for each gene set in GSEA and the statistical significance of the ES was estimated by an empirical permutation test using 2.000 gene permutations to obtain the nominal p-value.

### Quantitative realtime PCR (Q-RT-PCR)

Total RNA from 9 PCOS patients before and after treatment with pioglitazone was treated with DNAse I (USB, Cleveland, Ohio) and reverse transcribed to single-stranded cDNA using TaqMan reverse transcription reagents and random hexamer primers (Applied Biosystems). One patient was not included in the analysis because of a lack of RNA. TaqMan gene expression assays (Applied Biosystems) and TaqMan Universal Master Mix (Applied Biosystems) were used to quantify gene expression changes using Applied Biosystems Prism 7700 (Supplemental Table S9). β-actin (Applied Biosystems) was used as a housekeeping gene to normalize gene expression levels. To perform the most appropriate validation of microarray data, bioinformatic approaches such as NetAffx (www.Affymetrix.com), refseq (www.ncbi.nlm.nih.gov), and Ensembl (www.ensembl.org) were used to identify the TaqMan probe sequence for each gene, which has the highest similarity to the Affymetrix probe set (Supplemental Table S9).

## Supporting Information

Table S1 The ten most downregulated pathways analyzed with MAPPFinder 2.1.
(0.05 MB DOC)

Table S2 Ranking of the ten most downregulated gene sets analyzed with GSEA 2.0.1.
(0.05 MB DOC)

Table S3 The twenty most upregulated GO terms analyzed with MAPPFinder 2.1.
(0.07 MB DOC)

Table S4 Ranking of the twenty most downregulated GO terms analyzed with MAPPFinder 2.1.
(0.07 MB DOC)

Table S5 Ranking of the ten most upregulated pathways analyzed with MAPPFinder 2.1.
(0.05 MB DOC)

Table S6 The top-ten upregulated gene sets analyzed with GSEA 2.0.1.
(0.05 MB DOC)

Table S7 The twenty most upregulated GO terms analyzed with MAPPFinder 2.1.
(0.07 MB DOC)

Table S8 Ranking of the twenty most downregulated GO terms analyzed with MAPPFinder 2.1.
(0.07 MB DOC)

Table S9 Probe set and TaqMan assay for the 18 selected genes.
(0.08 MB DOC)
