# Supplementary material for: Pioglitazone Enhances Mitochondrial Biogenesis and Ribosomal Protein Biosynthesis in Skeletal Muscle in Polycystic Ovary Syndrome
Source: PLoS One. 2008 Jun 18;3(6):e2466. doi: 10.1371/journal.pone.0002466 (PMC2413008; doi:10.1371/journal.pone.0002466)
Supplement: Table S1 — The ten most downregulated pathways analyzed with MAPPFinder 2.1. (0.05 MB DOC) [file pone.0002466.s001.doc]

**Table S1**

**The ten most downregulated pathways analyzed** with MAPPFinder 2.1.

| MAPP Name | Changed (n) | Measured (n) | ON MAPP (n) | Changed (%) | Z Score | Permute p-value | FWER p-value |
| --- | --- | --- | --- | --- | --- | --- | --- |
| Blood group glycolipid biosynthesis lactoseries | 15 | 18 | 20 | 83.3 | 2.9 | 0.003 | 0.42 |
| Acetylcholine synthesis | 7 | 7 | 7 | 100.0 | 2.7 | 0.01 | 0.67 |
| Phosphatidylinositol signaling system | 74 | 123 | 133 | 60.2 | 2.5 | 0.01 | 0.90 |
| Biogenic amine synthesis | 12 | 15 | 15 | 80.0 | 2.4 | 0.01 | 0.95 |
| Tissue endocrine and CNS | 120 | 210 | 210 | 57.1 | 2.3 | 0.02 | 0.96 |
| Delta-notch netpath 3 | 52 | 85 | 85 | 61.2 | 2.2 | 0.03 | 0.99 |
| Keratan sulfate biosynthesis | 11 | 14 | 15 | 78.6 | 2.2 | 0.03 | 0.99 |
| B cell receptor netpath 12 | 91 | 158 | 158 | 57.6 | 2.1 | 0.04 | 1 |
| T cell receptor netpath 11 | 78 | 134 | 135 | 58.2 | 2.1 | 0.04 | 1 |
| Nuclear receptors | 25 | 38 | 38 | 65.8 | 2.1 | 0.05 | 1 |

A p-value < 0.05 and a fold change ≤ -1.05 were used as the criteria for gene expression changes in PCOS patients after pioglitazone treatment. The z-score is based on N = 4998 genes linked to a MAPP and R = 2460 of these genes meeting the criteria for change in expression. Changed (n): number of genes changed. Measured (n): number of genes measured on the chip. On MAPP (n): number of genes on the MAPP. Changed (%): Changed (n) divided by Measured (n). FWER p-value: Family Wise Error Rate.
