# Supplementary material for: Pioglitazone Enhances Mitochondrial Biogenesis and Ribosomal Protein Biosynthesis in Skeletal Muscle in Polycystic Ovary Syndrome
Source: PLoS One. 2008 Jun 18;3(6):e2466. doi: 10.1371/journal.pone.0002466 (PMC2413008; doi:10.1371/journal.pone.0002466)
Supplement: Table S2 — Ranking of the ten most downregulated gene sets analyzed with GSEA 2.0.1. (0.05 MB DOC) [file pone.0002466.s002.doc]

**Table S2**

Ranking of the ten most downregulated gene sets analyzed with GSEA 2.0.1.

| NAME | SIZE | ES | NES | NOM p-value | FDR q-value | FWER p-value |
| --- | --- | --- | --- | --- | --- | --- |
| GPCRS class A rhodopsin like | 134 | 0.47 | 1.81 | 0.0001 | 0.08 | 0.12 |
| GPCRDB class A rhodopsin like | 173 | 0.45 | 1.78 | 0.0001 | 0.06 | 0.17 |
| Monoamine GPCRS | 32 | 0.54 | 1.69 | 0.004 | 0.11 | 0.39 |
| GPCRDB other | 58 | 0.47 | 1.64 | 0.005 | 0.14 | 0.59 |
| Peptide GPCRS | 72 | 0.43 | 1.54 | 0.008 | 0.33 | 0.94 |
| ST GAQ pathway | 27 | 0.49 | 1.49 | 0.04 | 0.44 | 0.99 |
| Cell adhesion | 174 | 0.38 | 1.47 | 0.004 | 0.44 | 0.99 |
| ST GA13 pathway | 35 | 0.45 | 1.43 | 0.06 | 0.55 | 1 |
| MAPK cascade | 29 | 0.46 | 1.42 | 0.06 | 0.50 | 1 |
| Inflampathway | 29 | 0.46 | 1.39 | 0.08 | 0.58 | 1 |

All genes on the chip were ranked by difference in expression after pioglitazone treatment of PCOS patients using the t-test. An enrichment score (ES) was assigned to each gene, and the maximum ES (MES) was calculated for each gene set. NES: Enrichment score normalized for differences in gene set size. FDR q-value: False Discovery Rate. FWER p-value: Family Wise Error Rate.
