# Supplementary material for: Pioglitazone Enhances Mitochondrial Biogenesis and Ribosomal Protein Biosynthesis in Skeletal Muscle in Polycystic Ovary Syndrome
Source: PLoS One. 2008 Jun 18;3(6):e2466. doi: 10.1371/journal.pone.0002466 (PMC2413008; doi:10.1371/journal.pone.0002466)
Supplement: Table S3 — The twenty most upregulated GO terms analyzed with MAPPFinder 2.1. (0.07 MB DOC) [file pone.0002466.s003.doc]

**Table S3**

**The twenty most upregulated GO terms analyzed** with MAPPFinder 2.1.

| GO Name | GO Type | Changed (n) | Measured (n) | In GO (n) | Changed (%) | Z Score | Permute p-value | FWER p-value |
| --- | --- | --- | --- | --- | --- | --- | --- | --- |
| Ribonucleoprotein complex | C | 186 | 344 | 456 | 54.1 | 14.9 | <0.0005 | <0.0005 |
| Ribosome | C | 113 | 186 | 283 | 60.8 | 13.2 | <0.0005 | <0.0005 |
| Structural constituent of ribosome | F | 120 | 212 | 309 | 56.6 | 12.6 | <0.0005 | <0.0005 |
| Intracellular | C | 1663 | 6471 | 7205 | 25.7 | 11.6 | <0.0005 | <0.0005 |
| Intracellular organelle | C | 1413 | 5372 | 6012 | 26.3 | 11.2 | <0.0005 | <0.0005 |
| Organelle | C | 1413 | 5373 | 6013 | 26.3 | 11.2 | <0.0005 | <0.0005 |
| RNA binding | F | 203 | 493 | 545 | 41.2 | 10.9 | <0.0005 | <0.0005 |
| Protein biosynthesis | P | 210 | 521 | 644 | 40.3 | 10.7 | <0.0005 | <0.0005 |
| Cytoplasm | C | 804 | 2810 | 3067 | 28.6 | 10.4 | <0.0005 | <0.0005 |
| Protein complex | C | 522 | 1725 | 1994 | 30.3 | 9.5 | <0.0005 | <0.0005 |
| Macromolecule biosynthesis | P | 217 | 585 | 713 | 37.1 | 9.4 | <0.0005 | <0.0005 |
| Intracellular membrane bound organelle | C | 1210 | 4664 | 5138 | 25.9 | 9.2 | <0.0005 | <0.0005 |
| Membrane bound organelle | C | 1210 | 4665 | 5139 | 25.9 | 9.2 | <0.0005 | <0.0005 |
| RNA metabolism | P | 159 | 409 | 439 | 38.9 | 8.7 | <0.0005 | <0.0005 |
| RNA processing | P | 134 | 333 | 359 | 40.2 | 8.5 | <0.0005 | <0.0005 |
| Mitochondrion | C | 203 | 571 | 598 | 35.6 | 8.4 | <0.0005 | <0.0005 |
| Cellular metabolism | P | 1502 | 6094 | 6893 | 24.7 | 8.2 | <0.0005 | <0.0005 |
| Metabolism | P | 1586 | 6527 | 7383 | 24.3 | 7.8 | <0.0005 | <0.0005 |
| Oxidoreductase activity\, acting on NADH or NADPH\, quinone or similar compound as acceptor | F | 32 | 47 | 52 | 68.1 | 7.8 | <0.0005 | <0.0005 |
| NADH dehydrogenase activity | F | 30 | 43 | 48 | 69.8 | 7.7 | <0.0005 | <0.0005 |

A p-value < 0.05 and a fold change ≥ 1.05 were used as the criteria for gene expression changes in PCOS patients after pioglitazone treatment. The z-score is based on N = 13.443 genes linked to a GO term and R = 2884 of these genes meeting the criteria for change in expression. Changed (n): number of genes changed. Measured (n): number of genes measured on the chip. In GO (n): number of genes in the GO term. Changed (%): Changed (n) divided by Measured (n). FWER p-value: Family Wise Error Rate.
