# Supplementary material for: Pioglitazone Enhances Mitochondrial Biogenesis and Ribosomal Protein Biosynthesis in Skeletal Muscle in Polycystic Ovary Syndrome
Source: PLoS One. 2008 Jun 18;3(6):e2466. doi: 10.1371/journal.pone.0002466 (PMC2413008; doi:10.1371/journal.pone.0002466)
Supplement: Table S4 — Ranking of the twenty most downregulated GO terms analyzed with MAPPFinder 2.1. (0.07 MB DOC) [file pone.0002466.s004.doc]

**Table S4**

**Ranking of the twenty most downregulated GO terms analyzed** with MAPPFinder 2.1.

| GO Name | GO Type | Changed (n) | Measured (n) | In GO (n) | Changed (%) | Z Score | Permute p-value | FWER p-value |
| --- | --- | --- | --- | --- | --- | --- | --- | --- |
| Signal transducer activity | F | 981 | 2131 | 2705 | 46.0 | 7.4 | <0.0005 | <0.0005 |
| Intrinsic to membrane | C | 1411 | 3187 | 3855 | 44.3 | 7.1 | <0.0005 | <0.0005 |
| Integral to membrane | C | 1409 | 3183 | 3850 | 44.3 | 7.1 | <0.0005 | <0.0005 |
| Cell communication | P | 1210 | 2697 | 3262 | 44.9 | 7.1 | <0.0005 | <0.0005 |
| Signal transduction | P | 1100 | 2465 | 3006 | 44.6 | 6.5 | <0.0005 | <0.0005 |
| Membrane | C | 1767 | 4117 | 4861 | 42.9 | 6.4 | <0.0005 | <0.0005 |
| Plasma membrane | C | 701 | 1532 | 1626 | 45.8 | 5.9 | <0.0005 | 0.001 |
| Intrinsic to plasma membrane | C | 523 | 1114 | 1180 | 47.0 | 5.8 | <0.0005 | 0.001 |
| Integral to plasma membrane | C | 521 | 1110 | 1175 | 46.9 | 5.7 | <0.0005 | 0.001 |
| Receptor activity | F | 590 | 1280 | 1775 | 46.1 | 5.6 | <0.0005 | 0.001 |
| Cell-cell signaling | P | 243 | 480 | 517 | 50.6 | 5.4 | <0.0005 | 0.001 |
| Extracellular region | C | 453 | 965 | 1061 | 46.9 | 5.3 | <0.0005 | 0.001 |
| Development | P | 713 | 1590 | 1704 | 44.8 | 5.2 | <0.0005 | 0.002 |
| Cell adhesion | P | 264 | 532 | 563 | 49.6 | 5.2 | <0.0005 | 0.002 |
| Transmembrane receptor activity | F | 390 | 825 | 1268 | 47.3 | 5.1 | <0.0005 | 0.002 |
| Cation channel activity | F | 127 | 232 | 252 | 54.7 | 5.0 | <0.0005 | 0.003 |
| Ion transport | P | 312 | 649 | 706 | 48.1 | 4.9 | <0.0005 | 0.003 |
| Cell surface receptor linked signal transduction | P | 484 | 1058 | 1511 | 45.8 | 4.8 | <0.0005 | 0.003 |
| Cell differentiation | P | 196 | 389 | 423 | 50.4 | 4.7 | <0.0005 | 0.003 |
| Calcium ion binding | F | 348 | 739 | 819 | 47.1 | 4.7 | <0.0005 | 0.003 |

A p-value < 0.05 and a fold change ≤ -1.05 were used as the criteria for gene expression changes in PCOS patients after pioglitazone treatment. The z-score is based on N = 13.443 genes linked to a GO term and R = 5229 of these genes meeting the criteria for change in expression. Changed (n): number of genes changed. Measured (n): number of genes measured on the chip. In GO (n): number of genes in the GO term. Changed (%): Changed (n) divided by Measured (n). FWER p-value: Family Wise Error Rate.
