# Supplementary material for: Pioglitazone Enhances Mitochondrial Biogenesis and Ribosomal Protein Biosynthesis in Skeletal Muscle in Polycystic Ovary Syndrome
Source: PLoS One. 2008 Jun 18;3(6):e2466. doi: 10.1371/journal.pone.0002466 (PMC2413008; doi:10.1371/journal.pone.0002466)
Supplement: Table S5 — Ranking of the ten most upregulated pathways analyzed with MAPPFinder 2.1. (0.05 MB DOC) [file pone.0002466.s005.doc]

**Table S5**

**Ranking of the ten most upregulated pathways analyzed** with MAPPFinder 2.1.

| MAPP Name | Changed (n) | Measured (n) | ON MAPP (n) | Changed (%) | Z Score | Permute p-value | FWER p-value |
| --- | --- | --- | --- | --- | --- | --- | --- |
| IL-1 netpath 13 | 25 | 38 | 38 | 65.8 | 3.4 | 0.002 | 0.09 |
| B cell receptor netpath 12 | 79 | 158 | 158 | 50.0 | 2.9 | 0.003 | 0.45 |
| Phosphatidylinositol signaling system | 63 | 123 | 133 | 51.2 | 2.8 | 0.006 | 0.52 |
| Biogenic amine synthesis | 11 | 15 | 15 | 73.3 | 2.7 | 0.008 | 0.62 |
| MAPK cascade | 18 | 29 | 29 | 62.1 | 2.5 | 0.02 | 0.79 |
| Focal adhesion | 89 | 187 | 187 | 47.6 | 2.4 | 0.02 | 0.87 |
| Blood group glycolipid biosynthesis lactoseries | 12 | 18 | 20 | 66.7 | 2.4 | 0.03 | 0.89 |
| G protein signaling | 47 | 92 | 92 | 51.1 | 2.4 | 0.02 | 0.90 |
| MAPK signaling pathway | 77 | 161 | 162 | 47.8 | 2.3 | 0.03 | 0.94 |
| Apoptosis | 42 | 82 | 82 | 51.2 | 2.3 | 0.04 | 0.96 |

A p-value < 0.05 and a fold change ≥ 1.05 were used as the criteria for gene expression changes between PCOS patients and control subjects. The z-score is based on N = 4998 genes linked to a MAPP and R = 1952 of these genes meeting the criteria for change in expression. Changed (n): number of genes changed Measured (n): number of genes measured on the chip. On MAPP (n): number of genes on the MAPP. Changed (%): Changed (n) divided by Measured (n). FWER p-value: Family Wise Error Rate.
