# Supplementary material for: Pioglitazone Enhances Mitochondrial Biogenesis and Ribosomal Protein Biosynthesis in Skeletal Muscle in Polycystic Ovary Syndrome
Source: PLoS One. 2008 Jun 18;3(6):e2466. doi: 10.1371/journal.pone.0002466 (PMC2413008; doi:10.1371/journal.pone.0002466)
Supplement: Table S6 — The top-ten upregulated gene sets analyzed with GSEA 2.0.1. (0.05 MB DOC) [file pone.0002466.s006.doc]

**Table S6**

**The top-ten upregulated gene sets analyzed** with GSEA 2.0.1.

| NAME | SIZE | ES | NES | NOM p-value | FDR q-value | FWER p-value |
| --- | --- | --- | --- | --- | --- | --- |
| GPCRDB class A rhodopsin like | 173 | -0.41 | -1.66 | < 0.0001 | 0.52 | 0.48 |
| Monoamine GPCRS | 32 | -0.51 | -1.62 | 0.01 | 0.42 | 0.65 |
| GPCRS class A rhodopsin like | 134 | -0.40 | -1.60 | < 0.0001 | 0.34 | 0.72 |
| ST GAQ pathway | 27 | -0.50 | -1.55 | 0.02 | 0.44 | 0.90 |
| GH pathway | 27 | -0.47 | -1.44 | 0.05 | 0.92 | 1.00 |
| ST FAS signalling pathway | 61 | -0.39 | -1.44 | 0.02 | 0.79 | 1.00 |
| ST B cell antigen receptor | 39 | -0.42 | -1.39 | 0.06 | 1 | 1 |
| Nuclear receptors | 39 | -0.40 | -1.37 | 0.08 | 1 | 1 |
| Testis expressed genes | 61 | -0.37 | -1.35 | 0.07 | 1 | 1 |
| Peptide GPCRS | 72 | -0.36 | -1.35 | 0.06 | 0.97 | 1 |

All genes on the chip were ranked by difference in expression between PCOS patients and control subjects using the t-test. An enrichment score (ES) was assigned to each gene, and the maximum ES (MES) was calculated for each gene set. NES: Enrichment score normalized for differences in gene set size. FDR q-value: False Discovery Rate. FWER p-value: Family Wise Error Rate.
