# Supplementary material for: Pioglitazone Enhances Mitochondrial Biogenesis and Ribosomal Protein Biosynthesis in Skeletal Muscle in Polycystic Ovary Syndrome
Source: PLoS One. 2008 Jun 18;3(6):e2466. doi: 10.1371/journal.pone.0002466 (PMC2413008; doi:10.1371/journal.pone.0002466)
Supplement: Table S7 — The twenty most upregulated GO terms analyzed with MAPPFinder 2.1. (0.07 MB DOC) [file pone.0002466.s007.doc]

**Table S7: The twenty most upregulated GO terms analyzed** with MAPPFinder 2.1.

| GO Name | GO Type | Changed (n) | Measured (n) | In GO (n) | Changed (%) | Z Score | Permute p-value | FWER p-value |
| --- | --- | --- | --- | --- | --- | --- | --- | --- |
| Cell communication | P | 933 | 2697 | 3262 | 34.6 | 5.5 | <0.0005 | <0.0005 |
| Enzyme regulator activity | F | 222 | 560 | 597 | 39.6 | 5.0 | <0.0005 | 0.005 |
| Signal transduction | P | 847 | 2465 | 3006 | 34.4 | 4.9 | <0.0005 | 0.005 |
| Intrinsic to membrane | C | 1071 | 3187 | 3855 | 33.6 | 4.8 | <0.0005 | 0.007 |
| Integral to membrane | C | 1069 | 3183 | 3850 | 33.6 | 4.7 | <0.0005 | 0.007 |
| Guanyl nucleotide exchange factor activity | F | 54 | 106 | 114 | 50.9 | 4.7 | <0.0005 | 0.007 |
| Cellular component unknown | C | 255 | 666 | 768 | 38.3 | 4.6 | <0.0005 | 0.007 |
| Intracellular signaling cascade | P | 367 | 1007 | 1065 | 36.4 | 4.5 | <0.0005 | 0.02 |
| GTPase regulator activity | F | 108 | 254 | 265 | 42.5 | 4.3 | <0.0005 | 0.04 |
| Axonemal dynein complex | C | 8 | 8 | 11 | 100.0 | 4.3 | <0.0005 | 0.05 |
| Enzyme activator activity | F | 90 | 206 | 212 | 43.7 | 4.2 | <0.0005 | 0.06 |
| Protein binding | F | 1175 | 3572 | 3828 | 32.9 | 4.1 | <0.0005 | 0.11 |
| Membrane | C | 1343 | 4117 | 4861 | 32.6 | 4.0 | <0.0005 | 0.14 |
| Di-\, tri-valent inorganic cation transport | P | 51 | 108 | 119 | 47.2 | 3.9 | <0.0005 | 0.23 |
| Signal transducer activity | F | 719 | 2131 | 2705 | 33.7 | 3.9 | <0.0005 | 0.24 |
| Caspase activator activity | F | 8 | 9 | 9 | 88.9 | 3.8 | <0.0005 | 0.27 |
| Enzyme binding | F | 63 | 141 | 148 | 44.7 | 3.8 | 0.001 | 0.30 |
| Apoptotic protease activator activity | F | 9 | 11 | 11 | 81.8 | 3.7 | 0.001 | 0.31 |
| Cytoskeleton organization and biogenesis | P | 119 | 299 | 331 | 39.8 | 3.6 | 0.001 | 0.65 |
| Tissue morphogenesis | P | 7 | 8 | 9 | 87.5 | 3.5 | 0.001 | 0.73 |

A p-value < 0.05 and a fold change ≥ 1.05 were used as the criteria for gene expression changes between PCOS patients and control subjects. The z-score is based on N = 13.443 genes linked to a GO term and R = 1952 of these genes meeting the criteria for change in expression. Changed (n): number of genes changed. Measured (n): number of genes measured on the chip. In GO (n): number of genes in the GO term. Changed (%): Changed (n) divided by Measured (n). FWER p-value: Family Wise Error Rate.
