# Supplementary material for: Pioglitazone Enhances Mitochondrial Biogenesis and Ribosomal Protein Biosynthesis in Skeletal Muscle in Polycystic Ovary Syndrome
Source: PLoS One. 2008 Jun 18;3(6):e2466. doi: 10.1371/journal.pone.0002466 (PMC2413008; doi:10.1371/journal.pone.0002466)
Supplement: Table S8 — Ranking of the twenty most downregulated GO terms analyzed with MAPPFinder 2.1. (0.07 MB DOC) [file pone.0002466.s008.doc]

**Table S8**

**Ranking of the twenty most downregulated GO terms analyzed** with MAPPFinder 2.1.

| GO Name | GO Type | Changed (n) | Measured (n) | In GO (n) | Changed (%) | Z Score | Permute p-value | FWER p-value |
| --- | --- | --- | --- | --- | --- | --- | --- | --- |
| Ribosome | C | 73 | 186 | 283 | 39.2 | 13.1 | <0.0005 | <0.0005 |
| Structural constituent of ribosome | F | 76 | 212 | 309 | 35.8 | 12.4 | <0.0005 | <0.0005 |
| Ribonucleoprotein complex | C | 103 | 344 | 456 | 29.9 | 12.2 | <0.0005 | <0.0005 |
| Protein biosynthesis | P | 116 | 521 | 644 | 22.3 | 9.2 | <0.0005 | <0.0005 |
| Macromolecule biosynthesis | P | 122 | 585 | 713 | 20.9 | 8.6 | <0.0005 | <0.0005 |
| Mitochondrion | C | 118 | 571 | 598 | 20.7 | 8.4 | <0.0005 | <0.0005 |
| Cellular biosynthesis | P | 165 | 924 | 1073 | 17.9 | 7.9 | <0.0005 | <0.0005 |
| Cytoplasm | C | 401 | 2810 | 3067 | 14.3 | 7.8 | <0.0005 | <0.0005 |
| Biosynthesis | P | 177 | 1045 | 1205 | 16.9 | 7.4 | <0.0005 | <0.0005 |
| Intracellular | C | 794 | 6471 | 7205 | 12.3 | 7.3 | <0.0005 | <0.0005 |
| Organellar ribosome | C | 14 | 26 | 27 | 53.8 | 7.3 | <0.0005 | <0.0005 |
| Mitochondrial ribosome | C | 14 | 26 | 27 | 53.8 | 7.3 | <0.0005 | <0.0005 |
| Protein complex | C | 260 | 1725 | 1994 | 15.1 | 7.0 | <0.0005 | <0.0005 |
| Intracellular organelle | C | 673 | 5372 | 6012 | 12.5 | 7.0 | <0.0005 | <0.0005 |
| Organelle | C | 673 | 5373 | 6013 | 12.5 | 7.0 | <0.0005 | 0.001 |
| RNA binding | F | 96 | 493 | 545 | 19.5 | 6.9 | <0.0005 | 0.001 |
| Oxidoreductase activity\, acting on NADH or NADPH\, quinone or similar compound as acceptor | F | 19 | 47 | 52 | 40.4 | 6.8 | <0.0005 | 0.001 |
| Oxidoreductase activity\, acting on NADH or NADPH | F | 24 | 68 | 74 | 35.3 | 6.8 | <0.0005 | 0.001 |
| Small ribosomal subunit | C | 16 | 36 | 47 | 44.4 | 6.8 | <0.0005 | 0.001 |
| NADH dehydrogenase activity | F | 17 | 43 | 48 | 39.5 | 6.3 | <0.0005 | 0.01 |

A p-value < 0.05 and a fold change ≤ -1.05 were used as the criteria for gene expression changes between PCOS patients and control subjects. The z-score is based on N = 13.443 genes linked to a GO term and R = 1381 of these genes meeting the criteria for change in expression. Changed (n): number of genes changed. Measured (n): number of genes measured on the chip. In GO (n): number of genes in the GO term. Changed (%): Changed (n) divided by Measured (n). FWER p-value: Family Wise Error Rate.
