# Supplementary material for: Pioglitazone Enhances Mitochondrial Biogenesis and Ribosomal Protein Biosynthesis in Skeletal Muscle in Polycystic Ovary Syndrome
Source: PLoS One. 2008 Jun 18;3(6):e2466. doi: 10.1371/journal.pone.0002466 (PMC2413008; doi:10.1371/journal.pone.0002466)
Supplement: Table S9 — Probe set and TaqMan assay for the 18 selected genes. (0.08 MB DOC) [file pone.0002466.s009.doc]

**Table S9**

Probe set and TaqMan assay for the 18 selected genes.

| Probe set | Gene | TaqMan assay |
| --- | --- | --- |
| **218563_at** | NDUFA3 | Hs00831413_s1 |
| **202026_at** | SDHD | Hs00829723_g1 |
| **218190_s_at** | UCRC | Hs00203593_m1 |
| 228142_at | UCRC |  |
| **201134_x_at** | COX7C | Hs01595220_g1 |
| 213846_at | COX7C |  |
| 217491_x_at | COX7C |  |
| **210149_s_at** | ATP5H | Hs01046892_gH |
| 1555998_at | ATP5H |  |
| **208997_s_at** | UCP2 | Hs00163349_m1 |
| 208998_at | UCP2 |  |
| **219195_at** | PGC1-α | Hs00173304_m1 |
| 1569141_a_at | PGC1-α |  |
| **1555282_a_at** | PGC1-β | Hs00370186-m1 |
| 1563943_at | PGC1-β |  |
| 1553639_a_at | PGC1-β |  |
| **204652_s_at** | NRF1 | Hs00602161_m1 |
| 204651_at | NRF1 |  |
| 211279_at | NRF1 |  |
| 211280_s_at | NRF1 |  |
| 243190_at | NRF1 |  |
| **202753_at** | PSMD6 | Hs00207850_m1 |
| 1555884_at | PSMD6 |  |
| 232284_at | PSMD6 |  |
| 237240_at | PSMD6 |  |
| **222229_x_at** | RPL26 | Hs00864008_m1 |
| **201492_s_at** | RPL41 | Hs00606029_g1 |
| 1557448_a_at | RPL41 |  |
| 213898_at | RPL41 |  |
| 216215_s_at | RPL41 |  |
| **201257_x_at** | RPS3A | Hs00832893_sH |
| 200099_s_at | RPS3A |  |
| 212391_x_at | RPS3A |  |
| **202333_s_at** | UBE2B | Hs00163311_m1 |
| 202334_s_at | UBE2B |  |
| 202335_s_at | UBE2B |  |
| 211763_s_at | UBE2B |  |
| 224118_at | UBE2B |  |
| 228588_s_at | UBE2B |  |
| 239163_at | UBE2B |  |
| **226660_at** | RPS6kB1 | Hs00177357_m1 |
| 204171_at | RPS6kB1 |  |
| 211578_s_at | RPS6kB1 |  |
| **221539_at** | EIF4EBP1 | Hs00607050_m1 |
| 215482_s_at | EIF2B4 |  |
| **209429_x_at** | EIF2B4 | Hs00248984_m1 |
| 202288_at | FRAP1 |  |
| **215381_at** | FRAP1 | Hs00234522_m1 |

Probe sets with the highest similarity to the TaqMan assays according to NetAffx (www.Affymetrix.com), refseq (www.ncbi.nlm.nih.gov), and Ensembl (www.ensembl.org) are shown in bold.
